# Mast cell ontogeny: From fetal development to life‐long health and disease

**DOI:** 10.1111/imr.13191

**Published:** 2023-02-08

**Authors:** Shin Li Chia, Simran Kapoor, Cyril Carvalho, Marc Bajénoff, Rebecca Gentek

**Affiliations:** ^1^ Institute for Regeneration and Repair, Centre for Inflammation Research & Centre for Reproductive Health The University of Edinburgh Edinburgh UK; ^2^ Centre d'Immunologie de Marseille‐Luminy (CIML) Marseille France

**Keywords:** cancer, developmental programming, erythro‐myeloid progenitors, hematopoietic stem cells, mast cells, mastocytosis

## Abstract

Mast cells (MCs) are evolutionarily ancient innate immune cells with important roles in protective immunity against bacteria, parasites, and venomous animals. They can be found in most organs of the body, where they also contribute to normal tissue functioning, for example by engaging in crosstalk with nerves. Despite this, they are most widely known for their detrimental roles in allergy, anaphylaxis, and atopic disease. Just like macrophages, mast cells were conventionally thought to originate from the bone marrow. However, they are already present in fetal tissues before the onset of bone marrow hematopoiesis, questioning this dogma. In recent years, our view of myeloid cell ontogeny has been revised. We now know that the first mast cells originate from progenitors made in the extra‐embryonic yolk sac, and later get supplemented with mast cells produced from subsequent waves of hematopoiesis. In most connective tissues, sizeable populations of fetal‐derived mast cells persist into adulthood, where they self‐maintain largely independently from the bone marrow. These developmental origins are highly reminiscent of macrophages, which are known to have critical functions in development. Mast cells too may thus support healthy development. Their fetal origins and longevity also make mast cells susceptible to genetic and environmental perturbations, which may render them pathological. Here, we review our current understanding of mast cell biology from a developmental perspective. We first summarize how mast cell populations are established from distinct hematopoietic progenitor waves, and how they are subsequently maintained throughout life. We then discuss what functions mast cells may normally have at early life stages, and how they may be co‐opted to cause, worsen, or increase susceptibility to disease.

## 1 MAST CELL ONTOGENY

Mast cells are tissue‐resident innate effector cells of the myeloid lineage. They are critical for host defense against bacteria such as *Staphylococcus aureus* ^1^ and other pathogens like helminths.^2^, ^3^, ^4^ They also mediate protection from venoms produced by a range of animals, including snakes and bees.^5^, ^6^, ^7^ With this in mind, it is perhaps not surprising that mast cells are evolutionarily conserved. Cells with mast cell features are already present in tunicates,^8^, ^9^, ^10^ where they may have protective functions that are still relevant to modern mammals. They were first described by Paul Ehrlich in 1877 as cells filled with characteristic cytoplasmic granules.^11^ These granules were thought of as either remnants of phagocytosis, or signs of mast cells feeding or nourishing surrounding cells. The latter interpretation gave them their name from the German word “mästen.” Based on their localization and granule content, mast cells are typically classified as either mucosal (MTMCs) or connective tissue‐type mast cells (CTMCs), which in humans, respectively, express tryptase or tryptase and chymase.^12^, ^13^, ^14^ Recognized already in the 1970s, there is now growing appreciation that mast cell heterogeneity extends beyond this dichotomy. For example, connective tissue‐type mast cells share a core transcriptional program but differ between the skin, peritoneal cavity, and the esophagus, trachea, and tongue.^15^ Moreover, mast cell phenotypes are not rigid, but can change in inflammatory conditions.^16^, ^17^, ^18^, ^19^, ^20^, ^21^ Following their discovery, the original belief was that mast cells are of mesenchymal origin.^22^ This notion was first challenged 40 years ago with the demonstration that bone marrow (BM) transplanted into irradiated hosts can generate mast cells.^23^ Using “beige” mice that have distinctively large granules owing to a mutation, Kitamura, and colleagues were able to identify donor‐derived mast cells in recipient tissues. These findings were the first indication that mast cells are indeed hematopoietic in nature. This was confirmed in in vitro studies and parabiosis, an experimental approach in which two animals are surgically joined, resulting in a shared blood circulation.^24^, ^25^, ^26^ Based on these landmarking studies, the consensus became that mast cells originate from BM hematopoietic stem cells (HSCs). A similar BM‐centric view has also been held for other immune cells like macrophages, which were long thought to be exclusively derived from monocytes, despite evidence to the contrary. However, it is now abundantly clear that many tissue‐resident immune cell lineages, including macrophages and certain innate lymphocytes, first originate from fetal‐restricted progenitors. Over the life course, these fetal‐derived cells can be supplemented with BM‐derived ones. As we will discuss below, this developmental pattern also applies to mast cells.

### 1.1 Layered hematopoiesis

**FIGURE 1:**
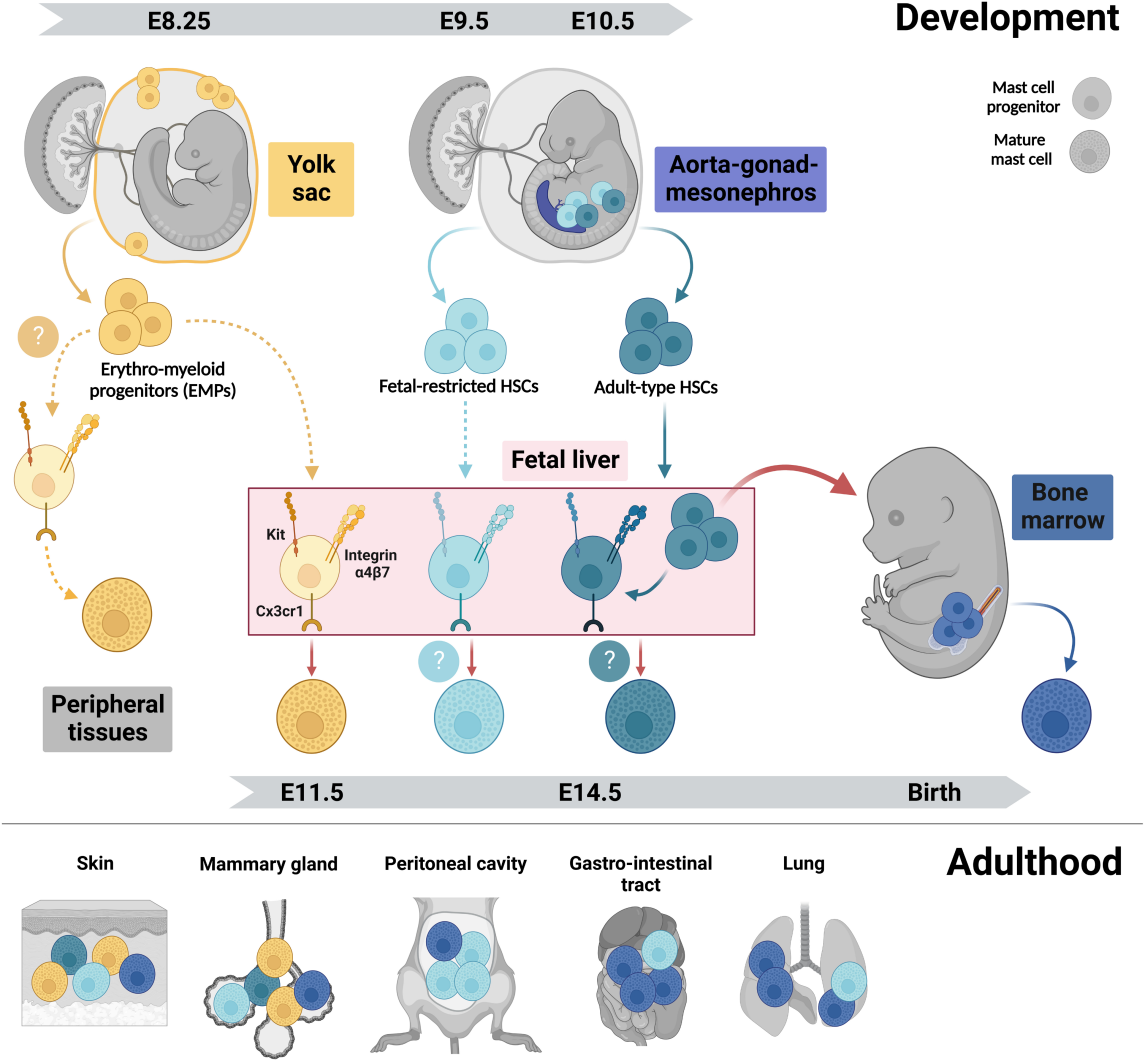
Mast cell development in the mouse. (Top) Different progenitors generating mast cells. The first mast cell progenitors arise from the erythro‐myeloid progenitors (EMPs) produced in the extra‐embryonic yolk sac starting at embryonic day 8.25 (E8.25). EMPs differentiate into mast cells via committed progenitors that may seed peripheral tissues directly from the yolk sac and/or through fetal liver intermediates. The intra‐embryonic aorta‐gonad‐mesonephros (AGM) region generates fetal‐restricted hematopoietic stem cells (HSCs) between E9.0 and E10.0 and adult‐type HSCs from E10.5 onwards. Both migrate to the fetal liver and may produce mast cells that supplement the first wave of EMP‐derived mast cells. Around birth, adult‐type HSCs colonize the bone marrow where they persist lifelong. Throughout life, bone marrow‐derived progenitors further complement mast cells notably at mucosal sites and in conditions of environmental perturbations. Mast cell‐committed progenitors express integrin α4β7, Cx3cr1, and Kit and undergo terminal maturation following recruitment into tissues. (Bottom) Origins of mast cells in adult tissues. In connective tissues like the skin and adipose tissues like the ones harboring the mammary glands, mast cells remain predominantly of fetal origins, with contribution from yolk sac EMPs and fetal‐restricted HSCs. In the lung and gut mucosa, fetal‐derived mast cells are largely replaced by bone marrow HSCs.

Hematopoiesis is a complex process that is initiated early in fetal development and involves several spatio‐temporally distinct (but overlapping) progenitor waves (Figure 1). The first hematopoietic progenitors are produced in the extraembryonic yolk sac (YS). In the mouse, so‐called primitive progenitors are made as early as day 7 of embryonic development (E7.0). These give rise primarily to erythrocytes and megakaryocytes, but also contribute to the very first macrophages.^27^, ^28^, ^29^, ^30^, ^31^ This is followed by the production of erythro‐myeloid progenitors (EMPs) starting at E8.25.^32^, ^33^ EMPs can still differentiate into cells of erythroid, megakaryocytic lineages, but also more readily produce different types of myeloid cells.^33^, ^34^ From E10.5 onwards, HSCs arise in the main arteries of the embryo properly. This occurs at several sites including the head, heart, placenta and vitelline, and umbilical veins,^35^, ^36^, ^37^, ^38^, ^39^, ^40^, ^41^, ^42^ though most notably, the aorta‐gonad‐mesonephros (AGM) region.^43^ Both, extra‐ and intra‐embryonic progenitors are generated from endothelial cells with hemogenic capacity in a process known as hemogenic‐to‐endothelial transition.^43^ HSCs transiently colonize and are thought to expand in the fetal liver, although this view has recently been challenged^44^ and hence, remains an area of ongoing debate. HSCs ultimately settle in the BM, where they persist lifelong. In addition to these well‐described waves, there are other HSC‐like progenitors generated (presumably) in the embryo proper starting at E9.0.^45^, ^46^ These are less well‐characterized, and since these waves overlap, their lineage output is difficult to disentangle from other progenitors. Alongside EMPs, these fetal HSCs are often referred to as transient‐definitive. This is because unlike primitive progenitors, they express the transcription factor c‐Myb,^47^, ^48^ but they cease to exist perinatally and are thus transient in nature. To account for these differences, we here refer to EMPs and fetal HSCs as fetal‐restricted, and to those HSCs that are produced in the AGM and take up residency in the BM as adult‐type HSCs.

### 1.2 Current understanding of mast cell origins

As mentioned above, work published in the 1960s‐1990s provided proof of concept that mast cells can originate from the BM. However, these seminal studies also already called into question whether the BM is the only source. It was observed that the rate of mast cell reconstitution from the BM is tissue‐dependent, but overall low. Indeed, in the pioneering work from Kitamura et al^23^ it took several months for the mast cell compartment along the gastrointestinal tract to be repopulated from grafted BM even in recipients conditioned by irradiation. Even after 6 months, very few if any donor BM‐derived mast cells could be detected in the skin of these animals. Furthermore, mast cells with metachromatic granules can already be found in fetal tissues, that is, before the onset of BM hematopoiesis.^49^, ^50^ In keeping with this, mast cell potential was identified in the extra‐embryonic yolk sac and fetal liver.^27^, ^49^, ^51^ To assess this, the Kitamura group adoptively transferred cells obtained from different fetal stages and sources into adult mast cell‐deficient W/Wv mice (see Box 2), either intravenously or directly into the skin.^51^ This revealed that between E9.5 and E11.5, the highest mast cell potential is found in the YS, followed by the fetal liver that predominates from E13.5 onwards.^51^ The presence of progenitors with mast cell potential in the YS and fetal liver was also later confirmed in more refined assays.^33^ Finally, work from the 1990 s described a population of lineage‐committed cells present in the fetal blood.^52^ These were phenotypically identified by high expression of Kit, low‐level expression of Thy1 and a low density of granules. They also express transcripts for three different mast cell proteases, Cpa3, Mcpt2, and Mcpt4. These Kit^high^ Thy1^low^ cells are first detected at E14.5, peak in numbers at E15.5 and decline towards birth. In vitro, they efficiently produce mast cell colonies, but not any other lineages. In vivo, they repopulate peritoneal mast cells upon intra‐peritoneal injection into deficient W/Wv recipients. Based on these features, they were considered circulating fetal mast cell progenitors or “pre‐mast cells” that have undergone partial differentiation prior to reaching their destination tissue. Alternatively, this population may represent mature fetal mast cells that are transiently present in the fetal circulation, which are terminally differentiated, but phenotypically distinct from their adult counterparts. Collectively, these findings showed that mast cells can in principle originate from fetal‐restricted progenitors found in the YS and fetal liver. However, they were based on either in vitro differentiation assays or adoptive transfer into hosts that were not age‐matched and either pre‐conditioned by irradiation or mast cell‐deficient. It thus remained unclear whether fetal progenitors indeed produce mast cells under physiologic conditions, and if mast cells originating from such fetal‐restricted progenitors persist postnatally.

**FIGURE 2:**
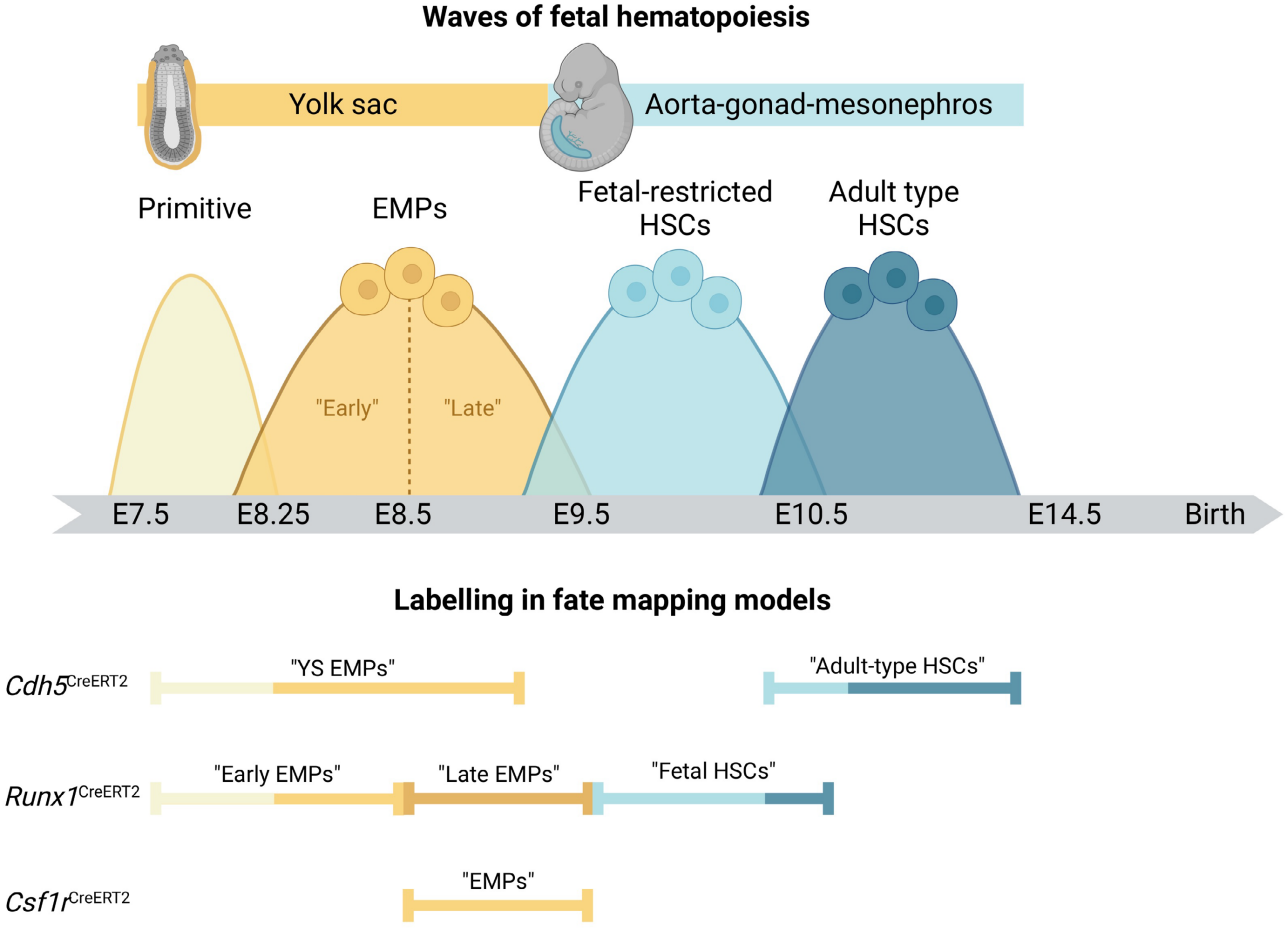
Waves of fetal hematopoiesis and their labeling using genetic fate mapping in mice. (Top) Overlapping waves of hematopoiesis during fetal development. Fetal hematopoiesis occurs in a rapid succession of several waves that partially overlap in space and time. The extra‐embryonic yolk sac first produces primitive progenitors that do not contribute to mast cells. This is followed by single, continuous wave of erythro‐myeloid progenitors (EMPs) that produce the first mast cells. At the time that EMPs production ceases, hematopoiesis commences intra‐embryonically in the aorta‐gonad‐mesonephros region. The first hematopoietic stem cells (HSCs) are fetal‐restricted but remain poorly defined. This is followed by the production of adult‐type HSCs that colonize the bone marrow perinatally. (Bottom) Labeling of distinct and overlapping progenitor waves using genetic fate mapping. Key models that have been used to study mast cell ontogeny are shown. They depend on label induction in either endothelial cells with hemogenic activity (*Cdh5*, encoding vascular E‐Cadherin), pan‐hematopoietic progenitors (*Runx1*, encoding a transcription factor essential for hematopoiesis), or myeloid‐committed progenitors (*Csf1r*, which encodes a growth factor receptor expressed by EMPs). Labeling is achieved through Cre‐mediated recombination resulting in expression of a fluorescent protein. Recombination is induced at specific developmental time points matching hematopoietic waves through administration of 4‐hydroxy‐tamoxifen (4OHT). Double quoted annotations indicate what each labeling approach has been interpreted as in the literature on mast cell ontogeny. However, as discussed in the main text and Box 1, EMPs likely constitute a continuous wave (see main text and labeling partially overlaps between waves and fate‐mapping models). This is illustrated by color‐coded bars. Since there is currently no single fate‐mapping model specific to either wave, data obtained in the different approaches should be interpreted in a complementary manner.

These questions were re‐addressed recently using genetic fate mapping, which can establish lineage relationships between progenitors and their progeny. Three different fate mapping approaches unequivocally established that in vivo and under physiological conditions, the first mast cells are produced from YS EMPs.^53^, ^54^ The frequency of EMP‐derived mast cells gradually declines over time, although sizeable populations are retained in adult connective tissues, such as the body skin, as well as adipose tissues. Simultaneously, the frequency of labeled mast cells increases with age in models that capture later hematopoietic waves. This demonstrated that the first wave of EMP‐derived mast cells is gradually diluted by mast cells originating from later progenitors. By far and large, the different models produced consistent data. However, different conclusions were drawn about the extent to which EMP‐derived mast cells persist postnatally, and about which progenitors constitute the second mast cell wave. We believe that these apparent discrepancies can be explained by the specifics and inherent limitations of the various fate‐mapping models used (discussed in Box 1). Consequently, we think existing data can be reconciled in an updated model of mast cell origins, whereby the first mast cells originate from a single wave of EMPs that are produced by the YS in a continuous manner (see Figure 2). These EMP‐derived mast cells are then supplemented with a second wave that originates predominantly from fetal‐restricted HSCs, but may also receive contribution from adult‐type HSCs. Considering that turnover from the BM is very low for mast cells in connective tissues populations,^23^, ^53^ such HSC‐derived mast cell progenitors might be recruited in the perinatal window prior to settling in the BM.

BOX 1 **What are the origins of the second mast cell wave?**

While several experimental strategies all identified yolk sac (YS) erythro‐myeloid progenitors (EMPs) as the source of the first mast cells, different conclusions were drawn about the origin of the second wave. Based on *Cdh5*‐CreERT2 fate mapping, we postulated that the second mast cell wave derives from adult‐type HSCs, whereas Li and colleagues concluded from the *Runx1*‐CreERT2 model that they originate from “late EMPs,” with a minor contribution from “fetal HSCs.”^54^ To consolidate these interpretations, it is important to consider that hematopoietic waves overlap spatially and temporally (see Figure 2). YS EMPs, the much more ill‐defined fetal‐restricted HSCs and adult‐type HSCs are all initiated in short succession between E8.25 and E10.5. However, each are produced for at least 1‐2 days and generate immune cell output for even longer. None of the currently available models can spatially distinguish YS from intra‐embryonic hematopoiesis, and all models available to study mast cell origins rely on a temporally defined distinction of different progenitors.

*Early, late, or one kind of EMPs?* The *Cdh5*‐CreERT2 and *Runx1*‐CreERT2 models exploit that the genes driving Cre recombinase are required for all (definitive) hematopoietic waves. Both rely on 4‐Hydroxy‐tamoxifen (4OHT) for label induction, the active metabolite that allows more precise timing than tamoxifen. However, 4OHT remains bio‐available for at least 24 hours.^246^ There is thus substantial overlap between progenitors labeled, for example, in *Runx1*‐CreERT2 mice pulsed at E7.5, E8.5, or E9.5. Moreover, the strict segregation of “early” and “late” EMPs (sometimes also referred to as EMP1 and EMP2) may simply reflect the timing of label induction, rather than biological differences. “Early” and “late” EMPs labeled in *Runx1*‐CreERT2 mice upon induction at E7.5 or E8.5 might thus simply correspond to capturing the two extremes of one continuous YS EMP wave. Indeed, intrinsic differences between “early” and “late” EMPs have not been described to our knowledge. On the contrary, EMPs obtained from the YS and fetal liver at different stages behave the same in clonogenic assays in vitro.^32^, ^247^ We thus think that rather than constituting a distinct second wave of “late EMP”‐derived mast cells, these still represent the first wave that is generated from EMPs produced in a continuous fashion in the YS.

*Fetal‐restricted or adult‐type HSCs?* The question then remains where the second wave of mast cells comes from that gradually dilutes EMP‐derived mast cells. Since bone marrow (BM) transplantation yields very limited mast cells even when transplanted into newborn mice,^54^ adult‐type HSCs either would need to seed tissues perinatally and bypass the BM, or they are not the major progenitor source. Both options seem plausible: Analysis of adult blood and immune cell lineages with known BM HSC origin confirms that induction of *Cdh5*‐CreERT2 at E10.5 does label adult‐type HSCs,^53^ nonetheless, this approach may also label fetal‐restricted HSCs. Conversely, E9.5 induction in the *Runx1* model may not only capture fetal‐restricted HSCs, but also some adult‐type ones. High levels of neutrophil labeling in 4 weeks old mice indicate that this is indeed the case, since neutrophils are short‐lived and of BM HSC origin at this stage. A very recent study supports a model whereby fetal‐restricted HSCs are the main source of the second mast cell wave. From a combination of genetic fate mapping, adoptive transfer, and in vitro differentiation assays, Yoshimoto and colleagues conclude that only the earliest HSCs found in the fetal liver give rise to mast cells, but not those present at a time when adult‐type HSCs dominate prior to BM colonization.^69^

It would follow that mast cells are established from YS EMPs, followed by fetal‐restricted HSCs. Adult‐type HSCs may contribute in a brief perinatal window without passing through the BM. While mast cells in connective tissues largely self‐maintain thereafter, mucosal mast cells receive further input from the BM after birth.

### 1.3 How do fetal progenitors generate mast cells?

In mice, granule‐containing, terminally differentiated mast cells can first be detected in the developing cornea at E12.5.^55^ In contrast, mast cells loaded with heparin‐containing granules have been reported only at E14.5‐E15.5 in the murine and rat skin.^49^, ^53^, ^56^ Moreover, others failed to detect them until E16.5 in mouse skin.^54^ This indicates that mast cells populate different organs at distinct developmental stages. Kinetics may even vary slightly for distinct regions of the same organ, such as the skin, which could explain differences between studies. As we will discuss later, this may reflect that mast cell colonization is driven by tissue‐ and stage‐specific demands, as has been discussed for macrophages.^57^ Lastly, it is possible that fetal mast cells carry out effector functions even when they do not yet have granules. Indeed, mast cells found in mesenteric lymph nodes of adult mice infected with the helminth *Trichinella spiralis* potently produce IL‐4 and IL‐6 despite being hypogranulated.^58^ During development, mast cells may thus be functional earlier than granule detection alone would suggest.

In adulthood, progenitors first commit to the mast cell lineage and then undergo terminal maturation in peripheral tissues. Similarly, EMPs produce macrophage‐committed progenitors (“pre‐macrophages”) that enter tissues via the blood stream and give rise to fetal macrophages. It is thus likely that the same applies to EMPs producing the first mast cells. Committed progenitors with an immature mast cell phenotype can be found in the circulation and peripheral tissues of developing mice and rats from E11.5 onwards.^54^, ^56^ These do not yet have granules but express cardinal markers of mast cells such as Kit, as well as several mast cell proteases, at least on transcriptional level.^54^, ^59^, ^60^ This indicates that lineage commitment indeed takes place prior to tissue colonization, while final maturation of fetal mast cells occurs in tissues.

EMP‐derived pre‐macrophages populate the fetus in a chemokine‐dependent process.^61^ This is at least partially mediated by interactions between Cx3cl1 (also known as fractalkine) and its receptor Cx3cr1,^62^ which is also expressed by fetal mast cells and their progenitors.^53^, ^63^ The Cx3cl1/Cx3cr1 axis may thus also be involved in the trafficking of mast cell progenitors to peripheral tissues. This could be addressed in *Cx3cr1* knockout mice. Considering that for macrophages, numbers in peripheral tissues are only transiently reduced early during development, and normalized later on, it will be important to also assess the relevance of this signaling axis for mast cells at the earliest stages possible, that is, when mast cells are normally detectable in substantial numbers in tissues.^62^ Mast cell progenitor recruitment likely also involves integrin signaling. Integrin α4β7 specifically is expressed by adult BM progenitors with mast cell potential^64^ and required for gut homing of mast cell progenitors.^65^ Indeed, integrin α4β7 is expressed by EMP‐derived progenitors that can be found in the fetal liver at E11.5.^53^, ^54^ These progenitors phenotypically still resemble EMPs,^66^ except for the lack of CD93 expression.^54^ Following in utero transplantation into the liver of age‐matched, unconditioned recipients, these EMP‐derived integrin α4β7^+^ progenitors readily produce mast cells detectable in the skin and peritoneal cavity by E18.5. Phenotypically similar fetal liver progenitors that lack integrin α4β7, on the other hand, do not give rise to mast cells. These data provide proof of concept that EMP‐derived fetal mast cell progenitors are marked by expression of integrin α4β7. However, integrin α4β7 is also expressed on progenitors for other immune cells, including BM‐derived progenitors for T cells and innate lymphoid cells, and is often thought of as a more general tissue homing marker.^67^, ^68^ The nature of the mast cell‐specific committed progenitor downstream of EMPs (and potentially other fetal‐restricted progenitors) thus remains to be fully elucidated. Moreover, it is unclear if integrin α4β7 is functionally required for tissue homing of these progenitors.

Another open question is whether mast cell progenitors can directly seed fetal tissues, as is the case for the first macrophages, or always require a fetal liver intermediate. Based on granule staining, terminally differentiated mast cells may appear after macrophages. It thus seems plausible that EMPs will first pass through the fetal liver before committing to the mast cell fate. In support of this notion, progenitors with mast cell potential can be found in the fetal liver from E11.5 onwards, and these progenitors give rise to mast cells when transferred into fetal or newborn recipients.^54^, ^69^ Nonetheless, this does not exclude that a direct route from the YS exists as well. Indeed, based on transplantation assays, mast cell potential is present in the YS until E13.5, and mast cells are in fact generated more efficiently from the YS than the fetal liver until E11.5.^51^ At E11.5, EMP‐derived mast cell progenitors expressing integrin α4β7 are also already present in the fetal circulation and body.^54^ Interestingly, those found in the limbs are transcriptomically very similar to those in the fetal liver at the same stage, yet do show some differences in gene expression, suggesting they have either started differentiating in situ and must have been recruited earlier, and/or they may have originated elsewhere. To delineate these options, one would need to compare mast cell progenitors from the different hematopoietic sites and peripheral tissues at various developmental stages in a comprehensive manner. Taken together, existing data are in line with YS EMP‐derived mast cell progenitors colonizing the developing fetus both directly and via the fetal liver.

Lastly, it is unclear which signals instruct fetal progenitors to commit to the mast cell lineage. They will likely involve stem cell factor (SCF) as well as IL‐3 and key transcription factors like Gata2, which are needed for mast cell specification from BM progenitors,^70^, ^71^, ^72^ but may also include additional, fetal‐specific factors. We also do not know how this core program is integrated with tissue‐specific signals to define mast cell (sub)populations. For EMP‐derived pre‐macrophages, acquisition of tissue‐specific transcriptional programs is initiated soon after their recruitment to peripheral tissues. This depends on unique transcription factors that are induced by micro‐environmental cues.^61^ Fetal mast cell specification may involve similar mechanisms. In line with this, human fetal mast cells show considerable heterogeneity across developmental stages (7‐17 weeks of gestation) and tissues analyzed, that is, liver, BM, thymus, spleen, and skin.^60^ These transcriptional differences parallel those seen in fetal macrophages.^60^ Nonetheless, differences likely also exist. For example, (pre‐) macrophages can be imprinted by environmental cues via phagocytic cargo, whereas mast cell progenitors or “pre‐mast cells” may respond to different signals, and/or sense these in a different way.

### 1.4 How are mast cells maintained postnatally?

Decades of work have established that progenitors with mast cell differentiation potential exist in the adult BM. In both mice^64^, ^73^ and humans,^74^, ^75^ BM HSCs produce mast cells in a stepwise process from multipotent (MPP) to lineage‐committed progenitors via common myeloid (CMP) and granulocyte‐monocyte progenitor (GMP) intermediates.^76^, ^77^ Within GMPs, bipotent progenitors (BMCPs) characterized by expression of Kit, CD34, CD16/32, and integrin α4β7 generate basophils and mast cells. Recent work that transcriptionally profiled BM progenitors on the single cell level found that BMCPs are segregated from GMPs by expression of *FcεR1a* and *Ms4a2*, which, respectively, encode the α and β subunits of the high‐affinity IgE receptor, FcεR1.^78^ Protein expression of FcεR1, on the other hand, appears to be variable for committed progenitors in peripheral tissues of mice and is also not found on human peripheral blood progenitors.^79^ How basophil and mast cell fates are separated within BMCPs is still not fully resolved.

Moreover, although the BM undoubtedly can produce mast cells, its actual contribution in vivo is limited under physiological conditions. As introduced above, mast cell replenishment from BM transferred into lethally irradiated mice is restricted to mucosal sites like the cecum and stomach, in which approximately 50% of resident mast cells are of donor BM origin after 8 months.^23^, ^54^ It has been suggested that this is due to tissue‐specific differences in radiosensitivity of mast cells. Skin‐resident mast cells, for example, are largely radio‐resistant,^80^ and there should thus be less need to replenish them from the BM. However, BM contribution is equally limited in “shielded” chimeras, in which most of the body is protected from irradiation,^53^ as well as in parabiotic animals that are not irradiated at all.^24^ Indeed, over a period of 4.5 months, only mast cells in the cecum, stomach, and spleen receive sizeable input from the parabiotic partner.^24^ Radiosensitivity alone can thus not explain the strikingly different turnover rates observed for connective and mucosal mast cells. While cell‐intrinsic difference may also contribute, environmental factors are likely key drivers of mast cell turnover. Mast cells residing in the intestinal mucosa, for example, are constantly exposed to food antigens and the microbiome. This could explain why they show the highest turnover from the BM, akin to intestinal macrophages.^81^ Supporting this notion, mast cells are strongly reduced in the intestinal mucosa of germ‐free mice.^82^ Dermal mast cells, on the other hand, do not receive considerable BM input, despite also populating a barrier site. They are, however, also reduced and appear less mature in the skin of germ‐free mice.^83^ It will thus be critical to decipher exactly which factors drive maintenance and replenishment of mast cells at different sites, as well as their initial colonization during development.

An interesting explanation for the low degree of BM turnover has been put forward in the 1980‐1990s, according to which mature mast cells may (actively) inhibit progenitor recruitment.^84^, ^85^ This was proposed to explain why peritoneal mast cells are only replenished from donor BM when mature mast cells are absent. Potential mechanisms were not explored, but this model seems to be in keeping with a recent study. Using genetic complementation at the morula stage, Weitzmann and colleagues generated animals in which mast cells are labeled by either RFP or YFP expression under control of *Mcpt5*‐Cre.^86^ In this approach, (ear) skin mast cells form mono‐colored clusters, indicating they have clonally expanded. At steady state, these clusters remain stable in size and distribution and are independent from BM input.^86^

Moreover, mast cells do not invade adjacent clusters. Mast cell density and distribution are thus tightly regulated. While the underlying mechanisms remain unknown, the availability of growth factors will likely play a role. The key growth factor for mast cells is SCF, the ligand for KIT, which is hence also known as KIT‐Ligand (KIT‐L). SCF is required for mast cell (progenitor) chemotaxis,^87^, ^88^ differentiation, adhesion,^89^, ^90^ proliferation, and survival by blocking apoptosis.^91^, ^92^, ^93^ Mutations that render KIT constitutively active cause aberrant mast cell expansion, as we will discuss below. On the other hand, mice with hypomorphic alleles of Kit are profoundly deficient in mast cells and have therefore long been used as models to study their functions. However, SCF is also important for other cell types and developmental processes, including hematopoietic stem and progenitor cells, melanocytes as well as primordial germ cells and spermatogonia in the testis. Kit‐dependent mice are therefore by no means mast cell‐specific and findings obtained in these models should be interpreted with caution (see Box 2). SCF exists in soluble and membrane‐bound forms, which are the result of alternative splicing of the same transcript.^94^ The membrane‐bound form is responsible for anchoring mast cells to the extracellular matrix, and it may also be the primary isoform required for mast cell development in vivo, since mice that have the soluble but not the membrane‐bound form are mast cell‐deficient.^95^ SCF can be produced by a range of non‐immune cells including fibroblasts and keratinocytes, and dermal mast cells are absent in mice in which SCF is specifically ablated in keratinocytes.^83^ Mast cells may also produce SCF themselves, at least in vitro.^96^ Exactly how SCF production is regulated remains unclear, but environmental factors like the microbiome likely play a role. Indeed, levels of SCF and consequently, mast cell numbers are lower in the skin of germ‐free compared to conventionally housed mice.^83^ While clearly important, availability of SCF and local mast cell density are unlikely the only factors which determine the extent and rate of their replenishment. Indeed, BM reconstitution is also surprisingly low even when recipients are used that are genetically mast cell‐deficient,^97^ in which it could be expected that the empty mast cell compartments are readily and rapidly repopulated from wild‐type progenitors. Replenishment from grafted wild‐type BM is not only very slow, it also does not restore wild‐type numbers.^86^, ^97^, ^98^ As these examples illustrate, we still do not fully understand which factors control mast cell numbers in healthy tissues.

BOX 2 **Mouse models to study mast cell functions**

A wide range of mouse models is available to study mast cell functions in physiological and pathological settings. These either lack all mast cells, some populations or certain mast cell effectors.

*Kit mutant mice*. Historically, mice were used that carry hypomorphic alleles of Kit, such as Kit^W/Wv^ and Kit^Wsh/Wsh^ mice. In these mice, mast cells are absent or reduced because they depend on Kit signaling for their growth and survival. However, Kit exerts pleiotropic functions both within and outside the hematopoietic system.^107^, ^248^ As discussed by many others, these models are therefore by no means specific for mast cells. For example, Kit^W/Wv^ mice have fewer neutrophils and basophils, are anemic and sterile. The Kit^Wsh/Wsh^ model, on the other hand, is neither anemic nor sterile but has a higher number of neutrophils and basophils. Additionally, Kit‐dependent models lack interstitial cells of Cajal and show abnormal pacemaker activity in the gastrointestinal tract. Results obtained from such models should be interpreted with caution, and their further use is discouraged.

*Kit‐independent models for mast cell deficiency*. Since the 2000s, the field has greatly benefited from the development of Kit‐independent mouse models, many of which are based on the Cre/LoxP system. In these models, Cre recombinase is under the control of genes expressed (almost) specifically by mast cells. Genes coding for mast cell proteases are commonly used, such as *Mcpt5* and *Cpa3*, the genes for mouse chymase and carboxypeptidase A3. Crossing these to the Cre‐responsive strains Rosa26^lsl‐DTA^ (for Diphtheria toxin alpha) or Rosa26^lsl‐hDTR^ (for human Diphtheria toxin receptor), respectively, results in constitutive or inducible depletion of mast cells.^249^ Alternatively, mast cell ablation can be achieved through Cre‐mediated depletion of anti‐apoptotic factors, such as Mcl‐1. This is the principle underlying “Hello Kitty” mice (*Cpa3*‐Cre; Mcl‐1^fl/fl^).^250^ Interestingly, an alternative approach to generating a Cre line using *Cpa3* resulted in a model that constitutively depletes mast cells without further crossing. In these “Cre‐Master” mice (*Cpa3* ^Cre/+^), knock‐in into the endogenous *Cpa3* locus results in Cre‐mediated genotoxicity.^251^ Other models were designed to achieve mast cell depletion in Cre‐independent approaches. For example, conditional depletion of can be achieved using “Mas‐TRECK” mice, in which DTR expression is under the control of intronic element of the *IL‐4* gene that normally drives IL‐4 expression in mast cells.^252^ In the “red mast cell and basophil” (RMB) mouse, DTR expression is under the control of the gene coding for the β chain of FcεR1 (*Ms4a2*),^253^ allowing for inducible mast cell depletion and hence the study of their functions, for example, at different disease stages. Collectively, these Kit‐independent models have allowed researchers to assess mast cell functions in a more specific manner. Indeed, using such models, many of the previous findings from Kit‐dependent models have been challenged.^254^ Nonetheless, these models are not without limitations. One main issue is that many of these also target basophils, albeit to varying degrees. Reduced basophil numbers are observed, for example, in Mas‐TRECK mice. RMB mice are an interesting model in this regard, since basophils recover much more quickly from Diphtheria toxin‐induced depletion than mast cells. This makes this model more mast cell‐specific at later stages. Other potential issues include off‐target effects or “Cre leakiness,” which can in principle be a concern for all Cre‐based approaches, as well as varying levels of depletion, certainly in inducible models. We therefore recommended using more than one model in a complementary manner where possible. Finally, most existing models do not differentiate (well) between (sub)populations of mast cells. While *Mcpt5*‐Cre mice are useful to distinguish functions of connective‐type from mucosal mast cell at least in the lung,^21^ RMB, Mas‐TRECK, and Cpa3‐based mice are less restrictive. It will therefore be important for the field to develop new models that allow more subset‐specific analysis of mast cells.

### 1.5 Non‐homeostatic mast cell replenishment

It has been proposed that BM progenitors are a primary source of mast cells following environmental perturbations, such as inflammatory insults.^99^, ^100^ In an early publication, donor BM‐derived mast cells appeared in the skin of lethally irradiated mice at sites that had been painted with methylcholanthrene, an irritant chemical with carcinogenic effects.^101^ Substantial influx of BM progenitors to the skin also occurs following application of 12‐O‐tetradecanoylphorbol‐13‐acetate (TPA), a small molecule drug originally derived from a plant that induces skin rashes.^86^ Within TPA‐treated inflamed skin, BM progenitors clonally expand and invade the territories of pre‐existing mast cells.^86^ Similarly, Dwyer and colleagues identified a population of progenitor‐like immature mast cells in nasal polyps of patients with chronic rhinosinusitis.^102^ These are distinct from circulating mast cell progenitors and appear highly proliferative. Cells with similar features were also found in fibrotic and asthmatic lung tissue.^102^ Finally, mast cell progenitors are also recruited to the airway mucosa upon allergic sensitization.^103^ These are thought to be BM‐derived, although this was not formally shown. Indeed, mast cell progenitors are also present in the spleen^104^ and murine adipose tissues,^105^ to which the latter are mobilized upon experimental myocardial infarction.^106^ As in mice, committed mast cell progenitors (or immature mast cells) have also been found at extra‐medullary sites in humans, including the spleen and a range of non‐lymphoid tissues, and they too can be recruited in inflammatory conditions.^10^ Overall, available data thus indicate that following perturbations, replenishment, or expansion of tissue‐resident mast cell populations can be achieved via several, complementary mechanisms. Progenitors can be mobilized from the BM or alternative sources, and/or recruited progenitors and (remaining) mature mast cells can (further) expand in the affected tissue by proliferation. The exact sources and cellular mechanisms underlying mast cell replenishment and hyperplasia in non‐homeostatic conditions likely depend on the nature of the insult.

In summary, mast cells are versatile immune cells that arise during fetal development and show considerable heterogeneity between and within tissues and in response to environmental perturbations. They are also heterogeneous with respect to their ontogeny. Connective tissue‐resident mast cells originate from YS EMPs and likely later fetal‐restricted progenitors. Once established, they self‐maintain independently from the BM. Mucosal mast cells, on the other hand, are partially replenished from the BM over time. Mast cell phenotypes are thus likely shaped by their origins and the microenvironments they encounter. As we will discuss in the following section, their functions may be adapted to the challenges of distinct life stages, and can potentially be co‐opted in pathological settings.

## 2 IMPLICATIONS OF MAST CELL ONTOGENY FOR LIFE‐LONG HEALTH AND DISEASE

Mast cells are best known for mediating allergy and anaphylaxis, but they also have other pathological roles. Aberrant expansion or activation of mast cells causes conditions known as mastocytosis and mast cell activation syndrome, and mast cells are also found associated with tumors. On the other hand, mast cells can also be protective, for example against *Staphylococcus*, honeybee, and snake venom.^1^, ^5^, ^6^, ^7^ Moreover, they have also been implicated in a variety of non‐immunological processes. These range from metabolism and neuronal crosstalk to tissue remodeling, wound healing, and angiogenesis,^107^ conditions that are biologically reminiscent of development. Here, we discuss how their ontogeny may be relevant to healthy development and pathology.

### 2.1 (Potential) functions of mast cells in early life

Fetal mast cells share their YS EMP origin with macrophages, which are known to have pivotal roles in development.^31^, ^32^, ^48^ Rather than waiting for adult‐type HSCs that become the dominant source of immune cells perinatally, fetal tissues are thus seeded with mast cells from the earliest possible source, suggesting they are required before birth. Indeed, mast cells appear to be functionally mature at fetal stages: In mice, mast cells detected as early as E12.5 in the cornea and E14.5 in the skin already contain characteristic granules.^53^, ^55^ At least on transcript level, fetal mast cells also express key effectors like proteases.^53^, ^54^, ^63^ This is also true for human fetal mast cells, which express, for example, *TPSAB1*, the gene encoding tryptase, and carry granules.^59^, ^60^, ^63^ While surface expression of FcεR1 is only upregulated perinatally,^53^, ^63^ fetal mast cells express other receptors through which they can sense the environment, such as the IL‐33 receptor.^53^, ^54^, ^63^ They can also respond to environmental signals and stressors already in utero.^63^ Producing functional mast cells at fetal stages may therefore serve at least two different purposes: Fetal mast cells could help protect from immunological threats before the adaptive immune system is set up, and/or they may support developmental processes.

#### 2.1.1 Do mast cells contribute to protective immunity at pre‐ and early postnatal stages?

In adults, mast cells have important functions in protective immunity from certain bacteria,^1^ helminths^2^, ^3^, ^4^ and animal venoms.^6^, ^7^ They are potent immune effectors that can rapidly respond to harmful stimuli owing to their intracellular granules loaded with pre‐formed effectors. Furthermore, mast cells precede the adaptive immune system both ontogenetically and phylogenetically. It seems plausible therefore that mast cells are also important for protective immunity in early life. One hypothesis is that they are present prior to birth primarily to prepare newborn mammals for immunological threats, for example from venomous animals or helminths. Specifically, it has been proposed that fetal mast cells may be primed in utero, for example through maternal IgE, allowing stronger and faster responses to harmful stimuli encountered after birth.^10^, ^63^, ^108^ If they were indeed critical for protective immunity in early life, it could be reasoned that prevalent infections constitute a strong evolutionary driver for maintaining mast cells. Helminth infections, for example, affect approximately one‐third of the total population, with strong regional bias towards tropic regions.^109^ Helminth infections are not commonly thought to be vertically transmitted to the fetus, however, transplacental infection has been described.^110^ Moreover, transmission through the mammary glands is a major route of infection after birth.^111^ Even where they are maternally restricted, helminth infections could be sensed by the developing fetus, for example by maternal cytokines.^112^ This is evidenced by a growing body of literature reporting protective effects of maternal helminth infections on offspring susceptibility to allergic disease. While seemingly at odds with potentiating postnatal immunity, the effects of maternal infections appear to be dependent on the phase of infection experienced during pregnancy, which for example for *Schistosoma mansoni* ranges from a predominantly type I and II to a regulatory response.^112^ It is thus conceivable that in utero priming of fetal (‐derived) mast cells by maternal helminth infections can indeed potentiate anti‐helminth immunity in infancy. Such a mechanism could be important considering that protective immunity against helminths is normally slow to develop.^109^

In addition to preparing for potential threats after birth, fetal mast cells may already have protective immune functions in utero. This might be of particular relevance for infections that can cause developmental defects, preterm delivery, or stillbirth, such as Group B *Streptococcus*.^113^, ^114^ Recent work demonstrated that mast cells help defend against these bacteria through the coagulation factor XIIIA.^115^ It seems possible that fetal mast cells may complement the action of the maternal immune system, and thereby safeguard fetal development. Although there is currently no experimental data showing this directly, human fetal mast cells acquire transcriptional signatures compatible with immune effector functions already from 10 weeks of gestation onwards.^60^, ^116^ Future studies should thus address if fetal mast cells help protect from maternally transmitted infections.

#### 2.1.2 Do mast cells support developmental processes?

Threats from parasites, other infectious agents or venomous animals may be common at least in wild animals and some human environments. Yet, one could argue that challenges intrinsic to development affect all individuals of a population. If fetal mast cells supported developmental processes to overcome such challenges, then these would be powerful evolutionary drivers for maintaining MCs in early life. Although the idea that mast cells support developmental processes has gained traction,^10^, ^11^, ^60^, ^117^ mice that lack mast cells develop normally without obvious defects, and mast cell deficiencies have not been reported in humans. The current consensus therefore is that mast cells are dispensable for development. Nonetheless, there is some evidence supporting developmental roles for mast cells. In rodents, they have been implicated in vasculature and nerve branching of the developing cornea.^55^ Mast cells colonize the developing cornea in two waves. The first mast cells concentrate around the corneal stroma from E12.5 onwards and disappear by the time of eyelid opening. A second wave of mast cells settles in the corneal limbus within the first postnatal week and stabilizes thereafter. These are recruited to the corneal limbus via a mechanism that depends on integrin α4β7 and Cxcr2.^55^ These kinetics mirror those of mast cells in other tissues, suggesting corneal mast cells may have similar developmental origins. At both stages, expansion of the local network to the required density may be achieved through in situ proliferation of mature mast cells. Of note, the area covered by blood vessels and the density of nerve fibers are both significantly reduced in newborn Kit^Wsh/Wsh^ mice that do not have corneal mast cells.^55^ This indicates that fetal mast cells mediate vascular and nerve branching in the developing cornea. The underlying mechanisms have not been explored in detail, but it was reported that corneal mast cells produce vascular endothelial growth factor (VEGF) and neurturin, which, respectively, have angiogenic and neurotrophic actions. In agreement with this, inappropriately high levels of mast cell‐derived VEGF promote neovascularization in the adult cornea, that is, excessive angiogenesis that can lead to impaired or lost vision.^118^

Mast cells can also be found in the developing brain of mice^54^ and rats,^119^ including the pre‐optic area.^120^ This brain region is essential for male copulatory behavior, which is marked by a higher synaptic density compared to females. Late fetal and early postnatal stages are critical for sexual differentiation in this region. At these stages, mast cell numbers are higher in the pre‐optic area of males, where mast cells are actively degranulating in response to estradiol released due to a surge in testicular hormones.^120^ Pharmacological inhibition of mast cells at late fetal stages dampens copulation behavior in adult male offspring, suggesting that masculinization is normally mediated by mast cells. Conversely, perinatal administration of exogenous estradiol or mast cell activation using the compound 48/80 both partially masculinize the copulation behavior of female mice. Mechanistically, mast cells modulate synaptic patterning indirectly through crosstalk with microglia.^120^ Mast cells thus regulate sexual differentiation in the brain during development in a hormone‐responsive manner and via mechanisms that involve other immune cells. Whether they also contribute to other developmental processes in the brain remains to be explored.

Developmental roles for mast cells may also extend beyond the fetal period, since many tissue maturation or remodeling processes continue or take place after birth. For example, female organs associated with reproduction undergo substantial tissue remodeling in preparation for pregnancy. In many aspects, the underlying biology of such tissue remodeling closely resembles fetal organogenesis. In the resting, non‐pregnant uterus, the blood vessels are thick‐walled. Early during pregnancy, these are converted into thin‐walled vessels with large lumens.^121^ This allows for effective exchange of oxygen and nutrients to meet the increased demands to the developing fetus. Of the innate immune cells that reside in the female reproductive organs, mast cells seem to be of particular importance for reproductive success. In the uterus, mast cell numbers increase during the receptive stage of the estrous cycle and early pregnancy, when they are in close association with the blood vessels at the implantation sites.^122^, ^123^ Mast cell‐deficient female mice display defective spiral artery remodeling and impaired implantation, collectively resulting in fetal growth restriction.^122^, ^124^, ^125^ Interestingly, mast cells appear to mediate these processes cooperatively with uterine NK cells, since both can (partially) compensate in the absence of the other.^125^ This functional redundancy may be a safety measure ensuring appropriate uterine remodeling. Mast cells have also been implicated in mammary gland remodeling at key developmental and reproductive stages. Mammary gland development is initiated at E10.5 in mice, but there are only a few rudimentary branches at birth. The glands remain relatively quiescent until puberty, when the ducts undergo rapid proliferation and branching, and epithelial cells form terminal end buds at the end of rudimentary ducts. Cells from these terminal end buds then invade the surrounding adipose tissue and form branches until the ductal tree is completed. During pregnancy, branching continues and ductal epithelium proliferates and differentiates to ultimately form the milk‐secreting alveoli. Weaning then triggers mammary gland involution and return to the pre‐pregnancy state. Mast cells localize to the terminal end buds of the mammary gland ducts during puberty, lactation, and subsequent involution.^126^, ^127^ Mast cell‐deficient Kit^Wsh/Wsh^ mice show a delay in pubertal ductal branching.^128^ Whether the transient nature of this defect is due to functional compensation by other cells is unknown. Likewise, the mechanisms by which mast cells may support branching morphogenesis in the mammary gland are not fully understood, but IL‐33 signaling, and mast cell proteases are likely involved. The plasminogen cascade and the signaling axis between IL‐33 and its receptor ST2 have been associated with both normal mammary gland development and tumorigenesis,^129^, ^130^ and IL‐33 signaling is important for mast cell survival and activation.^131^, ^132^ In the pubertal and post‐lactational mammary gland, mast cells also produce the protease plasma kallikrein, which activates the plasminogen cascade.^127^ Mast cell proteases may also enhance IL‐33 signaling^133^ and promote stromal remodeling via secretion of pro‐inflammatory cytokines like IL‐6^134^, ^135^ and IL‐13.^136^, ^137^ It is thus possible that effectors released by mast cells directly affect mammary gland remodeling, independently of other immune cells.^128^

Finally, single cell transcriptional profiling recently demonstrated that the earliest mast cells found in human fetuses express transcripts for pro‐inflammatory cytokines and chemokines involved in recruitment of endothelial cells.^60^ This has been interpretated as an indication that fetal mast cells are involved in tissue morphogenesis or angiogenesis also in humans.^60^ Although there is currently no further evidence for this, it has previously been speculated that human mast cell deficiencies may be embryonically lethal,^107^ a hypothesis that we think should be considered.

In conclusion, there is some evidence that mast cells do support healthy development at pre‐ and postnatal stages. Future studies should address if fetal(‐derived) mast cells are important for protective immunity before and shortly after birth. Moreover, we believe that evolutionary and developmental considerations provide a strong rationale to explore if and how mast cells support developmental processes more broadly. As the examples of the cornea, brain, and mammary gland illustrate, mast cells are likely involved in fine‐tuning such processes. The consequences of lacking mast cells may therefore be difficult to detect, in keeping with the overall normal appearance of mast cell‐deficient mice and the apparent absence of (symptomatic) human mast cell deficiencies. Moreover, even where mast cells did normally support tissue maturation, other cell types may be able to compensate in their absence. As suggested for the mammary gland, mast cell deficiency may thus result in subtle, transient phenotypes or developmental delays, rather than persisting overt defects. Addressing their roles in normal development will thus require hypothesis‐driven, in‐depth analyses of kit‐independent mast cell‐deficient models (see Box 2) at key stages relevant to the tissue of interest. Models allowing stage‐specific ablation may further aid in this. With such carefully designed approaches, we may ultimately be able to assess the relevance of findings obtained in mouse models for human development.

### 2.2 Abnormal mast cell development and pathology

Instead of supporting healthy development, mast cells can also become pathological. They can either rendered pathological themselves, through mutations or environmental programming, or their normal functions may be co‐opted for example by the tumor microenvironment. In the following, we discuss how mast cell ontogeny may be relevant to pathology.

#### 2.2.1 Mastocytosis and mast cell activation syndrome

Mastocytosis refers to a group of rare, heterogeneous disorders characterized by aberrant, clonal expansion of mast cells, which are accordingly classified as myeloproliferative neoplasms. Most cases are benign, but mastocytosis can also be associated with other hematological malignancies,^138^ or can itself become malignant. Symptoms are complex and reflect the broad spectrum of biological activities of the released mast cell effectors. They range from skin reactions like itching, an increased risk of anaphylaxis and general fatigue to gastrointestinal complications such as cramps, diarrhea, and nausea as well as pain in muscles and joints.

Different forms of mastocytosis are distinguished according to the affected tissues as well the age of onset and clinical course. In cutaneous forms, mast cell expansion is restricted to the skin. The first case of mastocytosis described as early as 1869 was *Urticaria pigmentosa*,^139^ a cutaneous form, although it was only recognized as a mast cell disease several years.^140^ In addition to the skin, systemic mastocytosis often also involves the BM and additional sited including lymphoid organs like spleen and lymph nodes and other internal organs like the gastrointestinal tract and liver. In some cases, symptoms associated with systemic disease like gastrointestinal cramping and anaphylaxis occur without apparent involvement of the BM.^141^ Cutaneous and systemic mastocytosis are further distinguished from mast cell malignancies.^138^, ^142^, ^143^ Different types of mastocytosis also show a remarkable distinct clinical course: Pediatric‐onset disease is predominantly cutaneous, tends to follow a milder clinical course and usually regresses spontaneously by adolescence. Adult‐onset disease, however, is more frequently systemic and does not normally regress. While most adult patients have stable disease with different severities, adult mastocytosis can also turn into more aggressive, systemic disease, and even malignancy,^144^, ^145^ which both have poor prognosis.^146^, ^147^ Although attempts at a better classification are made regularly,^148^ mastocytosis remains clinically complex, and curative treatments are currently lacking. There is thus currently an urgent clinical need to improve patient stratification and explore new therapeutic avenues.

The cause of mastocytosis is mutational hyperactivation of KIT, the tyrosine kinase receptor for stem cell factor, which mast cell development and survival depend on. These mutations are typically somatic, and germline mutations are very rare.^149^, ^150^, ^151^, ^152^, ^153^, ^154^, ^155^ They result in constitutive or ligand‐independent KIT signaling, leading to uncontrolled mast cell expansion. Auto‐activating *KIT* mutations are a shared feature of different types of mastocytosis.^156^ Most common is a substituting point mutation in codon 186 (D186V), which is found in 80% of adult patients.^157^ Although less abundant in these patients, this mutation still accounts for almost 40% of pediatric cases, and of the remaining pediatric patients, another 40% carry activating mutations in other regions of the gene.^156^, ^157^ This indicates that while the specific nature of the mutations may have some prognostic relevance, different mutation unlikely explain the distinct clinical courses of transient pediatric and chronic adult disease. Indeed, progress in our understanding of disease etiology on both, the cellular and molecular level notwithstanding, we still do not understand the biological differences underlying these striking differences.

One aspect that has received limited attention is the possibility that pediatric‐ and adult‐onset mastocytosis may have different cells of origin, specifically EMP‐like progenitors and HSCs. Indeed, persistence of adult‐ and spontaneous regression of pediatric‐onset disease resemble the normal kinetics of mast cell development that we have outlined above: In mice, the first, YS EMP‐derived mast cells are gradually replaced by mast cells originating from later progenitor waves.^53^, ^54^ These changes mainly occur from late fetal to early postnatal stages, which correspond to the time pediatric‐ and adult‐onset are clinically distinguished, and the age regression often occurs in pediatric‐onset patients. Intriguingly, activating *KIT* mutations have been described in HSCs and more committed downstream progenitors in the BM and peripheral blood of adult patients with systemic disease,^77^, ^158^, ^159^, ^160^, ^161^ but not pediatric patients. This supports the hypothesis that pediatric‐ and adult‐onset mastocytosis may have distinct cells of origin. To test this notion experimentally in mice, activating *KIT* mutations could be introduced into different mast cell progenitors using Cre recombinase technology, an approach that causes mastocytosis in vivo.^162^

Another condition caused by aberrant mast cell responses is known as mast cell activation syndrome (MCAS). This heterogeneous disease is characterized by recurring episodes of allergic and anaphylactic symptoms, which typically involve multiple organ systems, such as the skin, lung, heart, and gastrointestinal tract. Common symptoms include skin itching, swelling, and flushing, wheezing, abdominal pain, cramping, and diarrhea. These symptoms are driven by high levels of potent mast cell mediators,^163^ such as tryptase, histamine, leukotriene E4, and prostaglandin D2.^164^ MCAS can present in combination with mastocytosis and other mast cell‐related co‐morbidities such as Ehlers Danlos Syndrome, a rare genetic condition affecting connective tissues. Most cases of MCAS are idiopathic,^164^ however. Symptomatic MCAS episodes appear to be triggered by environmental exposures, for example to psychological stress,^165^ sudden temperature changes or odors and chemicals.^166^ In addition to triggering symptoms, environmental exposures may also contribute to MCAS etiology: A recent study has explored a possible link between MCAS and (acquired) chemical intolerance, and proposed that xenobiotic exposures also render mast cells hyper‐sensitive in the first place.^166^ This study suggested that mast cell sensitization may be the result of either isolated high‐dose exposures, or cumulative low‐grade exposure events. Since mast cells are long‐lived, both scenarios could induce long‐lasting changes, for example, through epigenetic mechanisms, thereby causing MCAS. As mast cells are already present and responsive from fetal stages onwards, these considerations may extend to the pre‐ and early postnatal period. Indeed, MCAS can also affect children, and it is therefore tempting to speculate that environmental exposures experienced in utero or shortly after birth contribute to MCAS etiology. Keeping in mind the normal developmental kinetics of mast cells outlined above, it will be important to delineate the relative contribution of fetal‐derived and adult mast cells to MCAS pathogenesis, as well as to decipher to what extent the underlying biology is shared or distinct.

#### 2.2.2 Mast cells as targets of developmental programming

Based on numerous epidemiological studies, it is now firmly established that adult health and disease have developmental origins.^167^ Exposure to adverse environments in utero or at early postnatal stages is associated with a wide range of non‐communicable diseases,^168^ many of which are characterized by inflammation and aberrant immune responses.^169^ Maternal infections and inflammation, psychosocial stress, nutrition, pollution, and chemical exposures are among the many early life stressors shown to increase the risk of developing such pathologies. This phenomenon is known as pathological or developmental programming. Their ontogeny may make mast cells particularly susceptible to developmental programming: They are already present and phenotypically plastic at fetal stages and can sense environmental perturbations.^63^, ^170^ They are also long‐lived and many fetal‐derived mast cells persist postnatally. Much like macrophages,^57^, ^171^ with which they share developmental origins, mast cells may thus be functionally altered by exposure to early life adversity and thereby mediate or worsen disease in later life.

Allergy is a cardinal mast cell‐mediated pathology with established roots in early life. Allergic symptoms are caused by aberrant activation and degranulation of mast cells in response to otherwise innocuous stimuli and range from localized swelling and itching to life‐threatening anaphylaxis.^172^ The incidence of allergy is on the rise, both in developed and developing countries.^173^, ^174^, ^175^ Yet, while the central role of mast cells in driving pathology has long been established,^172^ the etiology of allergy remains unclear. Sensitization to allergens may begin before birth.^176^, ^177^, ^178^, ^179^ Indeed, heredity of allergy is more strongly associated with maternal IgE compared to paternal IgE.^180^, ^181^ A recent study provided a biological basis of this phenomenon with the demonstration that fetal mast cells are sensitized by allergen‐specific maternal IgE that passes the placenta, resulting in hypersensitivity upon postnatal encounter with the allergen.^63^ This mechanism thus propagates maternal disease to the next generation and could explain the rapidly rising incidence of allergic disease. Additional stressors may also play a role in programming allergy in offspring. The rise in allergy incidence has also been associated with a reduced exposure to microorganisms in early childhood.^182^ In what is known as the hygiene hypothesis, it was postulated that reduced microbial exposure results in a functionally restricted, “inexperienced” immune system that may be biased towards type II responses.^183^ This view may be oversimplified, however, and it originally did not take into account the prenatal period, although prenatal exposure to microorganisms inversely correlates with the risk of developing atopy.^184^ It has since been proposed that restricted maternal microbial exposure also renders their offspring hypersensitive, and that this is at least in part mediated by effects on fetal immune cells or their progenitors.^185^ In keeping with the hygiene hypothesis, germ‐free mice have reduced mast cell numbers in the intestinal mucosa and are resistant to food allergy models.^82^ Future studies need to determine exactly how mast cells are shaped by fetal and newborn microbial exposure. They should also explore if and how mast cells are developmentally programmed by other stressors associated with allergy, such as pollutants,^186^ nutrition^187^, ^188^, ^189^ and psychosocial distress.^190^

A firm link with early life adversity has also been established for neuro‐developmental and psychiatric disorders,^191^ and immune dysregulation is involved in driving these pathologies.^192^, ^193^ Central nervous system mast cells may be involved. Indeed, mast cells have been linked to anxiety,^194^ and MCAS patients frequently report suffering from psychiatric symptoms.^195^, ^196^ As discussed above, MCAS may be triggered by cumulative exposures to environmental triggers and may hence represent another example of mast cell programming. Hyperactivation of meningeal mast cells and behavioral changes were also reported upon maternal separation, an animal model for early life stress. Specifically, maternal separation results in reduced sucrose preference, a symptom of depression in rodents.^197^ These effects are more pronounced in females than in males.^197^ Stress‐associated hyperactivation of mast cells may also play a role in neurodegenerative diseases.^198^, ^199^

Lastly, mast cells are also associated with the peripheral nervous system, for example in the gut.^200^ Early life adversity^201^ and mast cell‐neuronal interactions have been proposed as drivers of abdominal pathogenesis in gastrointestinal diseases like irritable bowel syndrome (IBS).^200^, ^202^ The combination of maternal separation and early weaning induces symptoms of IBS in pigs, and these correlate with increased numbers and activation of intestinal mast cells.^203^ Intestinal mast cells are also increased and hyperactivated in mice following maternal separation, and myenteric glia displayed a heightened responsiveness to histamine.^204^ Mast cell hyperactivation by stress is likely at least in part driven by the corticotrophin‐releasing hormone (CRH) axis that regulates physiological stress responses.^205^ Moreover, aberrant placental CRH signaling has been liked to neurodevelopmental alterations in the fetus,^206^ which could be driven by mast cells. Indeed, mast cells express receptors for CRH^207^, ^208^ that may act in an antagonistic fashion. Signaling via CRH1 receptors triggers mast cell degranulation,^205^ whereas signaling through CRH2 receptors does not.^209^ Mast cell hyperactivation may thus for example be mediated by a disbalance in CRH receptors, which could be epigenetically imprinted. Microglia are developmentally programmed by prenatal stress in this way,^210^ and similar mechanisms could thus apply to mast cells.

#### 2.2.3 Could ontogeny be the key to understanding the roles of mast cells in cancer?

Mast cells were first found associated with carcinoma by Paul Ehrlich.^211^ They are present in most solid tumors, where they may shape the tumor microenvironment and determine therapy response like other immune cells. Mast cells can affect tumor growth in several ways: They can promote angiogenesis through production of VEGF and Fibroblast Growth Factor 2 (FGF2), and indirectly through degradation of the extracellular matrix, which results in release of additional pro‐angiogenic factors.^212^ Matrix degradation also supports malignant tissue remodeling and tumor invasiveness.^213^ Mast cells can also directly impact malignant cells. For example, mast cell‐derived histamine and TNF‐α regulate cytokine production by tumor cells, and tryptase can inhibit melanoma proliferation.^214^ Finally, mast cells can promote either an immuno‐suppressive tumor environment^215^ or potent anti‐tumor immunity, for example by recruiting cytotoxic lymphocytes.^216^ Nonetheless, their roles in cancer have remained heavily debated. In clinical and experimental studies alike, mast cells have been associated with both, beneficial and detrimental outcomes. This is true often even for the same tumor types, including pancreatic,^217^, ^218^ lung,^219^, ^220^ colorectal^221^, ^222^ and prostate cancer^223^, ^224^, ^225^ as well as melanoma.^220^, ^226^, ^227^, ^228^, ^229^, ^230^ Other reports concluded they have no role at all.^231^, ^232^, ^233^

The literature on melanoma is particularly controversial. Pro‐tumorigenic roles for mast cells were reported in studies from the early 2000 s, which found a correlation between mast cell counts and poor prognosis.^234^, ^235^ These studies suggested that mast cells promote tumor angiogenesis through VEGF. Other studies reported anti‐tumorigenic roles for mast cells in melanoma. For example, one report found that low densities of tryptase‐ and chymase‐positive mast cells correlate with poor prognosis and a higher melanoma grade.^236^ The authors suggested that high concentrations of serine proteases in the tumor microenvironment might impair melanoma growth. Indeed, it was later shown that melanoma cells can take up tryptase via exosome trafficking.^214^ This led to cleavage of histone H3 and lamin B1 and extensive nuclear remodeling, ultimately leading to reduced tumor growth. Of note, cleavage of histone H3 and loss of lamin B1 also occur during cellular senescence,^237^, ^238^, ^239^ a process known to limit tumor progression. Finally, several mouse studies have found that the absence of mast cells has no effect on skin carcinogenesis or the growth of subcutaneously inoculated melanoma.^231^, ^232^, ^233^ They have thus concluded that mast cells are not essential for either melanoma growth or anti‐melanoma immunity, and that mast cells are rather inert “bystanders.” This interpretation remains difficult to reconcile with their (peri)tumoral enrichment, however, which suggests active recruitment. An alternative explanation is that melanoma‐associated mast cells comprise different populations with distinct and potentially antagonistic effects on tumor growth. Although the Cre/LoxP‐based models used in these studies represent a major improvement over traditional Kit‐dependent models (see Box 2), many target mast cells rather indifferently. Indeed, subcutaneous growth of B16 melanoma is unaffected in *Mcpt5*‐Cre:Rosa^DTA^ mice,^231^, ^232^ which lack all mast cells in the skin. Intriguingly however, B16 metastases are drastically reduced in the lung,^232^ where only connective‐type mast cells are absent, but the mucosal‐type population marked by expression of integrin α4β7 is retained.^21^ This highlights how taking the heterogeneity of tumor‐associated mast cells into consideration could unravel biological functions that may otherwise be masked.

A key determinant of the heterogeneous mast cell functions in the tumor microenvironment may be their ontogeny. Again, much can be learned from the comparison with what we know about macrophages in tumors. Macrophages are recruited either de novo from BM‐derived monocytes or as pre‐existing mature cells from surrounding tissues, which again can have different origins. Fetal‐derived macrophages are often recruited to tumors at early stages^240^ to promote their growth^240^, ^241^, ^242^ and metastatic spread.^243^ They can also selectively interact with stromal cells that have reactivated fetal features, generating an “onco‐fetal ecosystem” characterized by marked immuno‐suppression.^244^ Specifically, in hepatocellular carcinoma, fetal‐derived macrophages characterized by expression of folate receptor beta (FOLR2) promote vascularization, proliferation, and an immuno‐suppressive environment through selective interaction with endothelial cells expressing Plasmalemma Vesicle Associated Protein (PLVAP), a marker of fetal liver endothelium.^244^ Similar mechanisms may be at play in other tumor types.^244^, ^245^ Based on their shared ontogeny, it is thus tempting to speculate that similar considerations also apply to tumor‐associated mast cells. Future studies will need to address if they are recruited to tumors from different sources and at distinct stages, and whether such populations impact tumors in different ways.

## 3 OPEN QUESTIONS AND FUTURE DIRECTIONS

**FIGURE 3:**
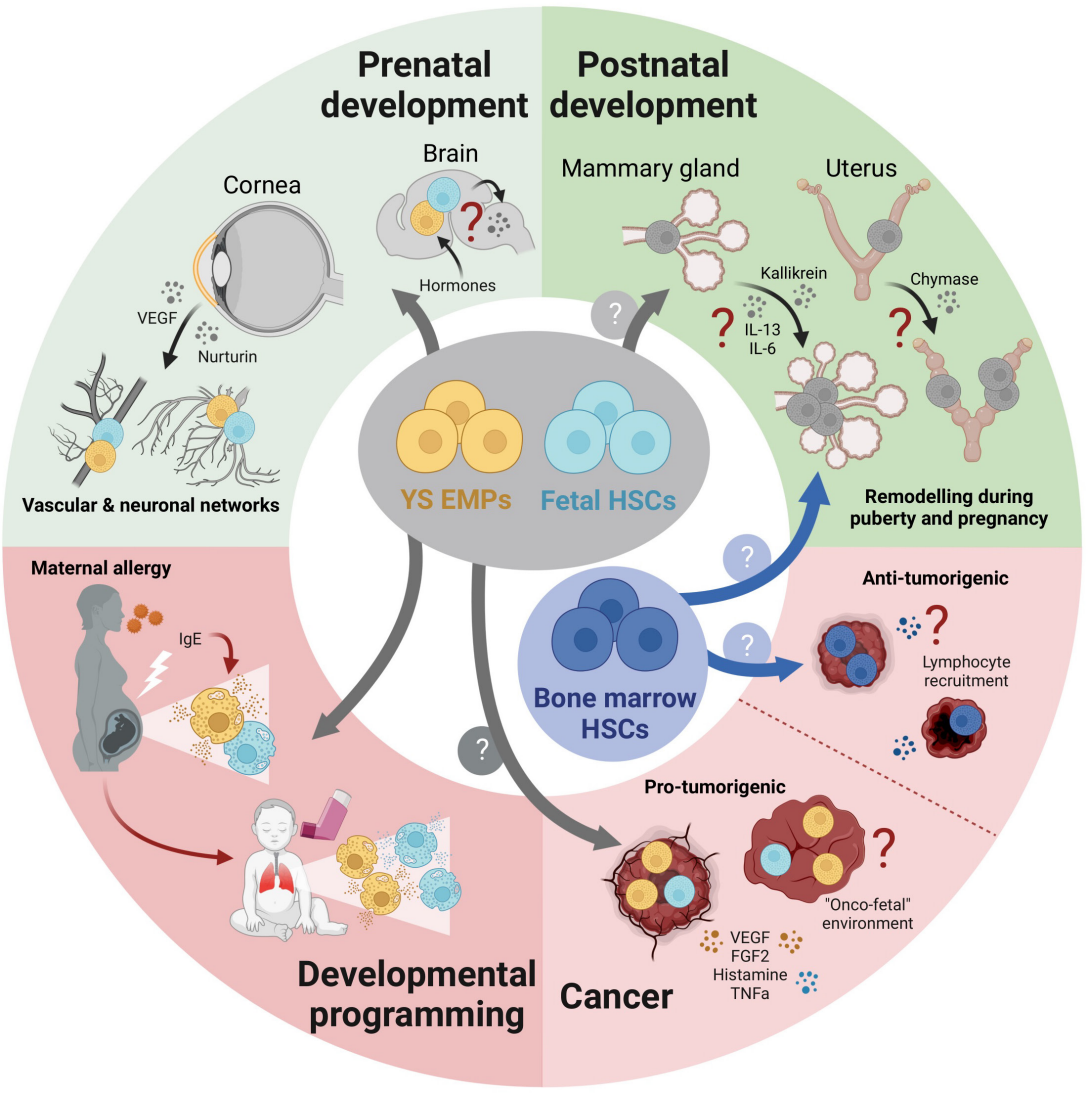
Functional implications of mast cell ontogeny for health and disease. (Centre) Mast cells may be generated from available progenitors to meet stage‐specific tissue demands. During normal development, they may support tissue remodeling processes. These physiological functions may be pathologically co‐opted by exposure to adverse stimuli during development, or for example by the tumor microenvironment. In disease, additional mast cells may be generated from the bone marrow and aberrantly imprinted. (Top) Mast cells support developmental processes taking place before, around, and after birth. In the developing cornea, they mediate fine‐tuning of vascular and neuronal networks. In the pre‐optic region of the brain of male mice, mast cells responding to hormones mediate synaptic patterning. During puberty and pregnancy, mast cells also support branching morphogenesis in the mammary gland and spiral artery remodeling in the uterus. (Bottom) Fetal mast cells are primed in utero by maternal allergy. This renders them hyperactivated, resulting in heightened offspring susceptibility to allergic disease. Mast cells with different developmental origins and microenvironmental imprinting could mediate their pro‐ and anti‐tumorigenic effects in cancer. Further explanations in the main text.

Our insight into mast cell biology is ever evolving. Advances in mouse models not only helped clarify their roles in a range of pathologies but also enabled the discovery of previously unappreciated mast cell functions. Through deep phenotyping, (single cell) transcriptomics, and proteomics, it has become abundantly clear that these cells are more heterogeneous than what is captured by the classical dichotomy of mucosal‐ and connective tissue‐type mast cells. These approaches have also enabled studying human mast cells with unprecedented resolution. We also now have an overhauled understanding of mast cell development. Mast cells are already present in the fetus, where they can sense and respond to environmental signals and thus, appear functionally mature. They are long‐lived, and most mast cell populations found in healthy connective tissues are established in the pre‐ or early postnatal periods and subsequently self‐maintain with site‐specific, but overall low input from the BM. These developmental dynamics have far‐reaching implications for mast cell biology in health and disease (see Figure 3): Their presence at early life stages suggests that mast cells may be needed during development. Their early life origins and longevity also make mast cells highly susceptible to genetic and environmental insults, which may render them pathological. Much like for macrophages, mast cell identity, and functions are thus likely shaped by their ontogeny, that is, the combination of their cellular sources and differentiation trajectories, the time they have spent in the tissue, the cumulative (micro)environmental signals they are exposed to during their lifespan, and any (epi)genetic changes resulting from these exposures. We believe that studying mast cells within this framework will help resolve persisting controversies and unravel the many aspects of their biology that remain poorly understood.

### 3.1 What is the relative contribution of different progenitors to mast cell (sub)populations?

While it is now firmly established that the first mast cells originate from EMPs produced in the extra‐embryonic YS, it is less clear to what extent these and subsequent progenitor waves contribute to the various mast cell compartments throughout life. Existing data from several fate mapping systems are best reconciled in a model in which most mast cells found in adult connective tissues originate from fetal‐restricted progenitors, with a sizeable contribution of YS EMPs. The remainder of connective as well as a proportion of mucosal tissue‐resident mast cells originates from either fetal‐restricted or adult‐type HSCs, or a mixture of both. To distinguish these possibilities, additional fate‐mapping models are required that better differentiate these successive hematopoietic waves. Future studies on their origins will also need to better appreciate mast cell (sub)populations, such as those in the lung marked by presence or absence of integrin α4β7.

Progenitors with mast cell potential are also present in mouse and human BM, but their contribution is tissue‐ and potentially environment‐specific. At steady state, most mast cell populations in mouse connective tissue are only minimally supplemented from the BM. While mucosal sites recruit more BM progenitors, the overall contribution during adult homeostasis remains considerably lower than for many other tissue‐resident lineages. At this point, we do not know exactly which signals drive BM recruitment at homeostasis. Considering that the abundance of BM‐derived mast cells is highest in the gut and lung mucosa, the microbiome may be a factor. On the other hand, skin‐resident mast cells are largely self‐maintained with neglectable BM input. It is therefore important to comprehensively (re)‐assess the contribution of BM progenitors to mast cells across the lifespan and sites, again taking into account subpopulations that may exist even within the same organ. Beyond the steady state, we should determine to what extent mast cells are generated de novo from the BM in response to infection or acute inflammation, whether BM‐derived mast cells persist following resolution of these insults and how they compare to pre‐existing mast cells. Similar considerations apply to chronic inflammatory conditions and cancer. Finally, it will be invaluable to expand on pioneering (single cell) transcriptomics studies in humans, using, for example, deep sequencing for somatic mutations in mitochondrial or nuclear DNA, technologies that provide a readout for cellular origins and lineage relationships.

### 3.2 When, where, and how are mast cells specified from fetal progenitors?

Despite continued progress in deciphering mast cell development, we still do not fully understand when, where, and how fetal (‐derived) mast cells are specified from their progenitors. This is in part because the kinetics with which mast cells and their progenitors colonize fetal tissues are incompletely resolved. Mast cells can first be detected in humans from the 8th gestational week onwards. Relative to the length of gestation, this is earlier than in rodents, where the first granule‐containing mast cells have been identified at E12.5, more than halfway through fetal development. In both humans and rodents, mast cells also seem to colonize different sites with distinct kinetics. These kinetics could potentially reflecting stage‐specific demands for mast cells. To better understand these, we thus need to comprehensively map when mast cells appear across fetal tissues and developmental stages using a combination of transcriptomics, immune phenotyping, and imaging approaches.

Mast cell progenitors appear in peripheral tissues before mature mast cells, indicating that fetal progenitors commit to the mast cell lineage prior to entering tissues and undergo further maturation there, much like what has been shown for adult progenitors. Although the core transcriptomic program that defines mast cells has been identified,^15^ we however do not know which signals instruct progenitors to initiate this lineage‐specific program. Considering their heterogeneity, it is likely that tissue‐ or population‐specific signatures are superimposed onto this core mast cell program during in situ maturation. Future work will need to identify the underlying molecular mechanisms, the tissue‐specific cues driving this, and the ways in which these are sensed and integrated by mast cells. We believe much can be learned from the comparison with macrophage specification, work on BM progenitors as well as the growing body of literature on human mast cell development. Finally, it is also still uncertain if there is a direct route for mast cell progenitors from the YS to peripheral tissues, like for some macrophages, or if instead, a fetal liver step is always required. There is evidence for both scenarios, and it is possible therefore that mast cell progenitors first follow a direct route from the YS and later are recruited from the fetal liver. However, this remains to be experimentally demonstrated.

### 3.3 Do mast cells have developmental functions? Are these co‐opted in pathology?

Their “layered” ontogeny suggests that mast cells may be recruited from available hematopoietic progenitors to meet the demands of specific developmental stages. It is thus conceivable that mast cells may have different functions throughout life. Before the adaptive immune system is established, one such demand is to protect the developing individual from pathogens and other immunological challenges, such as venoms. At least in the wild, these still pose a frequent threat to mammals. If mast cells were important for protective immunity in the pre‐ and perinatal period, this could be a reason why they are already present in the fetus, and why these cells have been maintained during evolution. In our view, an even more compelling reason would be if mast cells were involved in processes intrinsic to development. Although they are currently thought to be dispensable for normal development, there is evidence that they do contribute to tissue remodeling processes in the fetal cornea and pubertal mammary gland. It remains to be confirmed if these processes are truly mast cell‐dependent, and whether mast cells more broadly support tissue maturation and remodeling. However, in keeping with this idea, data obtained in Kit‐independent mouse models also found a role for mast cells in artery remodeling in the pregnant uterus.

Whatever roles mast cells may play in development and normal tissue functioning, it seems possible that these can be co‐opted in pathological conditions. In turn, their pathological actions, for example, in allergy, atopic disease, and cancer may “echo” their physiological functions. For example, sensitization of fetal mast cells by maternal IgE results in an increased allergen sensitivity postnatally. This exaggerated responsiveness represents a case of developmental programming but may also reflect a beneficial role for mast cells during normal development, which could prepare for rapid responses to harmful stimuli after birth, provided that fetal(‐derived) mast cells are appropriately primed in utero. Finally, it is possible that mast cells established from different sources at distinct life stages may retain functional differences into adulthood. These may be cell‐intrinsic, due to distinct environments experienced over their lifetime, or a combination of both. Such differences may be subtle or not at all apparent at steady state, however, they may become functionally relevant in non‐homeostatic conditions such as wound healing or cancer.

## AUTHOR CONTRIBUTIONS

The authors jointly wrote the manuscript and designed figures.

## CONFLICT OF INTEREST STATEMENT

The authors declare no competing interests.

## Data Availability

Data sharing not applicable—no new data generated.
